# Teaching therapy-oriented pharmacology within a medical curriculum

**DOI:** 10.1186/1471-2210-11-S2-A35

**Published:** 2011-09-05

**Authors:** Georg Wietzorrek

**Affiliations:** 1 Division of Molecular and Cellular Pharmacology, Innsbruck Medical University, 6020 Innsbruck, Austria

## Background

Pharmacology has been known to medical students as a hard-to-study subject that includes learning by heart hundreds of receptors and pathways, mechanisms of action, substances and side effects in a very limited time. Once graduated, young MDs are confronted with applied pharmacology without being properly prepared choosing and prescribing the right drug in the proper dose to individual patients.

## Methods and results

My new approach in teaching pharmacology focuses on the application of pharmacology in up-to-date therapy, stressing information relevant for decision-making and prescription. Exam questions contain patient cases to test prescribing and decision-making abilities. My approach in teaching pharmacology and clinical pharmacology within the curriculum with special focus in therapy was honoured by the students with two “Professor of the Term” awards and excellent ranking by the students and with outstanding results in the evaluation programme conducted by Innsbruck Medical University. The interest of the students in pharmacology grew, 116 students chose to attend extra classes in pharmacology beyond the curriculum during the last semester.

